# SOCIAL ACTIVITIES AND DEPRESSIVE SYMPTOMS AMONG OLDER ADULTS IN MEXICO: IMPLICATIONS OF GENDER AND PHYSICAL HEALTH

**DOI:** 10.1093/geroni/igz038.787

**Published:** 2019-11-08

**Authors:** Maria Monserud

**Affiliations:** University of Houston, Houston, Texas, United States

## Abstract

Studies in developed countries indicate that social activities can make a difference in mental health in later life. Yet, research on potential benefits of social activities for older adults in developing countries, including Mexico, has been scarce. This study uses the two most recent waves (2012, 2015) of the Mexican Health and Aging Study to investigate the impact of social activities on depressive symptoms among older men (n = 4, 749) and women (n = 6,527), aged 50+, in Mexico. The results of Ordinary Least Squares regressions indicate that it is important to differentiate among specific social activities in later life. Particularly, not only group-based but also solitary social activities were predictive of better mental health. Moreover, the findings demonstrate several gender differences and similarities. Participation in clubs, communication with relatives and friends, physical exercise, and watching television were beneficial for mental health among men, whereas volunteering, playing games, and making crafts were associated with fewer depressive symptoms among women. At the same time, reading as well as doing household chores were related to better mental health among older Mexicans, regardless of gender. Furthermore, this study shows that self-reported health, functional limitations, chronic conditions, and frequent pain might shape the implications of social activities for depressive symptoms among older adults in Mexico. The insights from this study can be helpful for intervention programs that are being developed to promote benefits of group-based and solitary social activities for mental health among older men and women with different levels of physical health.

